# A Novel SXXLF Motif in the FXR N‐Terminal Domain Mediates Coregulator and Interdomain Interactions

**DOI:** 10.1002/cbic.202500967

**Published:** 2026-09-10

**Authors:** Priscilla Villalona, Thilini Pulahinge, Tracy Yu, Jordan Wenning, Sabab Hasan Khan, Crawford Joseph Frisbie, Jill Magafas, Addison Barbe, C. Denise Okafor

**Affiliations:** ^1^ Department of Biochemistry and Molecular Biology The Pennsylvania State University University Park Pennsylvania USA; ^2^ Department of Chemistry The Pennsylvania State University University Park Pennsylvania USA

## Abstract

The nuclear receptor (NR) superfamily is comprised of ligand‐regulated transcription factors that contain an intrinsically disordered domain at the amino‐terminal end, known as the N‐terminal domain (NTD). While this poorly conserved domain is known to possess ligand‐independent activation function (AF‐1), few NTD functions are conserved between NRs. Identified roles in other receptors include androgen receptor (AR), estrogen receptor (ER), and mineralocorticoid receptor (MR). Here, we aim to define the function of the NTD of the farnesoid X receptor (FXR), a crucial regulator of lipid and bile acid metabolism. We show that the NTD engages in interdomain contact with other FXR domains. We also observe that the NTD interacts directly with coregulator proteins. Using mutagenesis, mammalian two‐hybrid assays, mammalian one‐hybrid assay, and molecular dynamics (MD) simulations, we identify and validate a novel SXXLF motif in the NTD which mediates interactions with both coregulators and the ligand binding domain. Mutation of the motif induces large changes in conformational and allosteric coupling in FXR. Our study identifies a new nuclear receptor‐interacting motif that modulates the transcriptional activity of FXR.

## Graphical abstract

Farnesoid X receptor (FXR)‐N‐terminal domain (NTD) regulates transcriptional activity through interdomain communication with the LBD and is also involved in co‐activator recruitment. The SENLF motif is the first defined functional element within the FXR‐NTD and mediates both NTD–LBD interaction and selective co‐activator engagements to drive NTD‐mediated transcriptional activity.

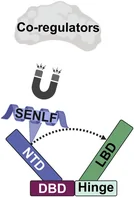

## 1 Introduction

In humans, the nuclear receptor (NR) superfamily comprises 48 ligand‐regulated transcription factors involved in many physiological processes, including metabolism, reproduction, homeostasis, and development [1]. Following the discovery of the first NRs four decades ago [2], distinct mechanisms of action observed in NRs identified four classes: Type I NRs are found in the cytoplasm, and when activated, they bind to their response elements on DNA as homodimers [1, 3]. Type II NRs are typically located in the nucleus, bound with corepressor proteins that are exchanged for coactivators upon ligand binding [1, 3]. The NRs in this group also form heterodimeric complexes with the NR retinoid X receptor (RXR) [1, 3]. Then, Type II and Type III NRs share the trait of residing in the cytoplasm but form homodimers rather than heterodimers [1, 3]. Finally, Type IV NRs can bind to their response elements as monomers [3].

The farnesoid X receptor (FXR, NR1H4) is a member of the NR superfamily [2] and is regulated by bile acids [4]. While it is classified as a Type II NR, found in the nucleus and forming a heterodimer with RXR [5], we also recently showed that FXR activates transcription as a homodimer [6]. FXR is involved in a wide array of biological processes, including bile acid homeostasis, glucose metabolism, and lipid metabolism [4, 7, 8]. FXR is encoded by FXRα, which gives rise to four isoforms (FXRα1, FXRα2, FXRα3, and FXRα4), resulting from alternative splicing and different promoter usage [7]. The isoforms are differentially expressed, with the first two moderately expressed in the ileum of the small intestine and in the adrenal glands, and the last two isoforms are mostly expressed in the ileum, with moderate levels in the kidney [4, 9].

Like other NRs, FXR has a modular structure composed of four domains: the N‐terminal domain (NTD), the DNA‐binding domain (DBD), a flexible hinge region, and lastly the ligand‐binding domain (LBD) [1, 10]. The NTD is a predominantly disordered domain in NRs, including FXR, and includes an activation function (AF‐1) region where coregulators can bind [1, 10]. Following the NTD is the DBD, which contains two zinc‐coordinated fingers that interact with specific DNA sequences known as FXR response elements (FXRE) recognized by the DBD [1, 10]. The LBD is usually formed by 11 helices and four beta strands that arrange to create a three‐layer α‐helical sandwich structure, with the hydrophobic amino acids at the core comprising the ligand‐binding pocket (LBP) [1, 10]. The C‐terminal helix (H12), along with H3 and H4, comprises the activation function 2 (AF‐2) surface, which plays a role in ligand‐induced coregulator recruitment [1, 10]. For FXR, many studies have characterized the LBD and DBD of the receptor, expanding our understanding of their functions [11, 12].

For the specific regulation of genes, NRs interact with coregulatory proteins. Coregulators can directly interact with NRs to activate or repress NR target genes [13]. Coactivators contain a conserved motif called the NR box (LXXLL, X = any amino acid) that interfaces with the AF‐2 region of NRs [13]. Coregulator interactions are also stabilized by a “charge clamp,” formed by electrostatic interactions between the LXXLL‐containing motif and conserved basic and acidic amino acids located on H3 and H12, respectively [13, 14]. Coregulators mediate cell‐ and ligand‐specific gene regulation via various mechanisms [13]. For example, some members of the steroid receptor coactivator (SRC) family possess intrinsic histone acetyltransferase activity (SRC1 and SRC3). This enzymatic activity is responsible for chromatin remodeling during gene activation [13, 15].

Several FXR‐interacting coregulators have been reported. When activated by ligands, FXR can interact with the transcriptional coregulator p300 [16]. For the expression of the small heterodimer partner (SHP), p300 acetylates both FXR and the histones at the promoter region of the SHP gene [16]. When p300 is downregulated, this decreases FXR presence in the promoter regions, leading to a decrease in SHP expression [16]. The p160/SRC family of coregulators interacts with the LBD of FXR, increasing its ability to activate its target genes [17]. They can also serve as linkers to other chromatin remodeling enzymes for transcriptional regulation [18]. Another coactivator, peroxisome proliferator–activated receptor‐γ coactivator (PGC‐1α), has been shown to influence FXR activity in two pathways [19]. The first pathway involves the activation of PPARγ and HNF4α, which then enhances FXR gene transcription [19]. In the second pathway, PGC‐1α interacts with the DBD of FXR to target FXR genes [19]. Also, MED1, a subunit of the mediator complex, plays a crucial role in NR activity, including for FXR [20].

The NTD of FXR and other NRs is highly disordered, making it challenging to use in structural studies to understand its function. Other NR NTDs have been found to participate in interactions with other binding partners and engage in interdomain crosstalk [21]. In the estrogen receptor (ER), the AF‐1 region, which is a site for ligand‐independent coregulator binding, has been found to interact with GRIP1 and SRC1α, members of the p160 coactivator family. In GST pull‐down assays, researchers identified regions of the AF‐1 that are important for binding to GRIP1 and SRC1α [22]. Through NMR and circular dichroism (CD) studies, it was shown that the NTD of both ERα and ERβ is unstructured in solution. However, the TATA‐box binding protein (TBP) has been demonstrated through surface plasmon resonance and CD to bind to the NTD of Erα, suggesting that upon TBP binding, the NTD undergoes structural changes, as observed by a reduction in random coil content [23]. Other NRs have shown that their NTDs participate in coregulator interactions. The NTD of the androgen receptor (AR) has been shown to interact with SRC1 in a ligand‐dependent manner [24]. It has also been observed that the NTD of rat mineralocorticoid receptor (MR) is capable of binding to p300 [25]. The NTD of Nurr1 has also been shown to interact with SRC1 through the AF‐1 region [26].

In addition to interactions with coregulatory proteins, studies have also provided evidence of interdomain interactions in NRs involving the NTD. For instance, in the AR, the AF‐1 domain was found to contain coregulator‐like interaction motifs, such as FXXLF and WXXLF, which participate in androgen‐dependent NTD and AF‐2 binding (for FXXLF). The WXXLF sequence binds in regions outside the AF‐2 in the LBD [27]. In the MR, the NTD has been reported to participate in interdomain interaction with the LBD as a result of strong agonist binding [28]. In the glucocorticoid receptor (GR), the N‐terminal region of the coactivator TIF2 binds to the NTD/AF‐1 region of the receptor, leading to conformational changes in the receptor [29]. In the progesterone receptor (PR), interdomain contacts between the AF‐1 and AF‐2 regions were observed when the receptor was not bound to DNA [30]. These studies have presented evidence that the NTD of NRs serves a function in the intrinsic activity of their receptors, whether this is through interacting with coregulator proteins or participating in interdomain NTD–LBD contacts. Apart from NTD‐specific interdomain interactions, other types of interdomain interactions, e.g., DBD‐LBD, have been reported in FXR [31], PPARγ, RARβ [5], HNFα [32, 33], and AR [34].

The role of the NTD in FXR has not been fully explored, specifically whether it participates in interdomain contacts and/or interacts with coregulators. Using luciferase reporter assays, mammalian two‐hybrid assays, mammalian one‐hybrid assays, and molecular dynamics (MD) simulations, we provide evidence for the role of the NTD in the FXRα1 isoform by elucidating its interdomain contacts and interactions with coregulators. We show that an SENLF motif in the NTD mediates the observed interactions. As this is the first report of an SXXLF NR‐interacting motif, these findings suggest that there may be undiscovered motifs involved in critical NR functions.

## 2 Results

### 2.1 The FXR NTD Interacts With Other FXR Domains

**FIGURE 1:**
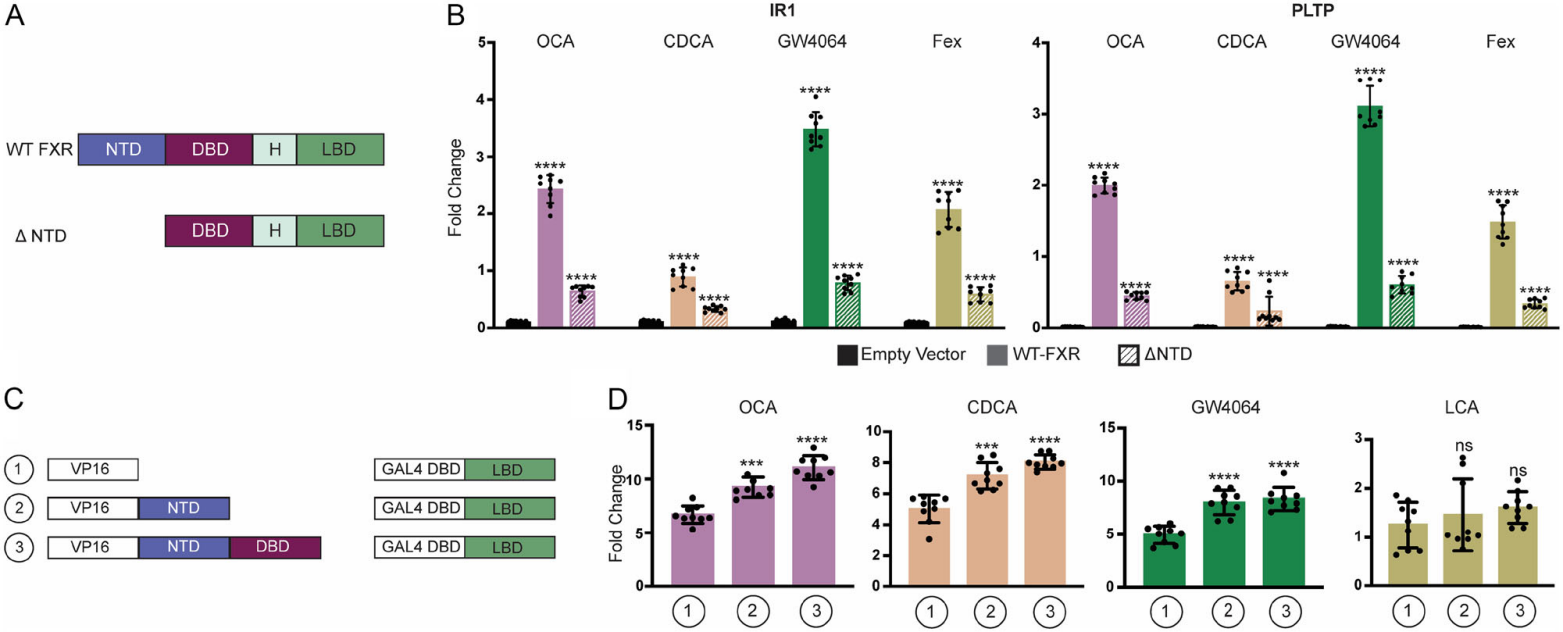
Effect of NTD deletion on FXR activity. (A) Schematic representation of WT‐FXR and ΔNTD. Domains are colored as follows: NTD in purple, DBD in maroon, hinge (H) in teal, and LBD in green. (B) Luciferase assays testing the effect of the NTD deletion on FXR activity. (C) Schematic representation of the hybrid constructs used in the M2H assay to assess the interdomain NTD–LBD contacts using the following constructs: NTD and NTD‐DBD fused to the VP16 individually. The LBD of FXR is fused to the GAL4‐DBD, giving GAL4‐DBD‐FXR‐LBD. (D) Luciferase assays using the M2H constructs to test interdomain contact between the NTD and LBD. With each additional domain of FXR, the fold change increases relative to the empty VP16 control with GAL4‐LBD. Error bars for luciferase assay: standard deviation. (B) Two‐way ANOVA and (D) one‐way ANOVA, *p* < 0.001 (***), (****) *p* < 0.0001.

The NTD of NRs is known to possess ligand‐independent activation function. In FXR, it is not known how the absence of the NTD impacts transcriptional activity. To assess the impact of the NTD on FXRα1 function, we generated a construct with the NTD removed (ΔNTD) (Figure 1A). Using two luciferase reporter constructs driven by FXREs, IR1‐*luc* and PLTP‐*luc* (see Section 4), we measured and compared transcriptional activity in response to ligand treatment in wild‐type FXR (WT‐FXR) and ΔNTD constructs. Four FXR agonists were tested: chenodeoxycholic acid (CDCA), fexaramine, obeticholic acid (OCA), and GW4064. Compared to WT‐FXR, a significant decrease in response (>50%) is observed in ΔNTD for all ligands (Figure 1B). This loss of activity confirms that the NTD is a major contributor to transactivation in FXR. A similar effect was observed in other NRs, such as the human ER [35], AR [36], and PR [37], when the NTD was fully or partially removed.

Previous work from our group reported interdomain interactions between the DBD and LBD of FXR. These interactions, observed using an mammalian two‐hybrid (M2H) assay, only occurred when the LBD was attached to the hinge domain [31]. We sought to investigate whether a similar M2H assay using the NTD would reveal interdomain interactions with other FXR domains. To determine whether the NTD interacts with the FXR‐LBD, two fusion protein constructs were generated: VP16‐NTD, with the FXR‐NTD fused to the VP16 activation domain, and GAL4DBD‐FXR‐LBD, with the FXR‐LBD fused to the GAL4‐DBD (Figure 1C). When the VP16‐NTD and GAL4DBD‐FXR‐LBD plasmids are co‐transfected and treated with FXR ligands, a significant increase in luciferase expression is observed, compared to the control group (empty VP16 and GAL4DBD‐FXR‐LBD) (Figure 1D). This observation confirms an interaction between the NTD and LBD. Interestingly, this increase is only observed with FXR agonists CDCA, OCA, and GW4064. The weak agonist LCA did not produce an increase in fold change over the control (Figure 1D).

To determine whether other FXR domains influence the NTD–LBD interaction, we created an additional construct: VP16‐NTD‐DBD (Figure 1C). The presence of the DBD led to a small but significant increase in fold change over the VP16‐NTD construct when cells are treated with OCA, CDCA, and GW4064 (Figure 1D). This suggests that under certain conditions, the DBD may enhance the NTD–LBD interaction. These experiments confirm that the FXR NTD is capable of interactions and binds with the LBD both in isolation and in the intact receptor.

### 2.2 The FXR NTD Interacts With NR Coregulators

**FIGURE 2:**
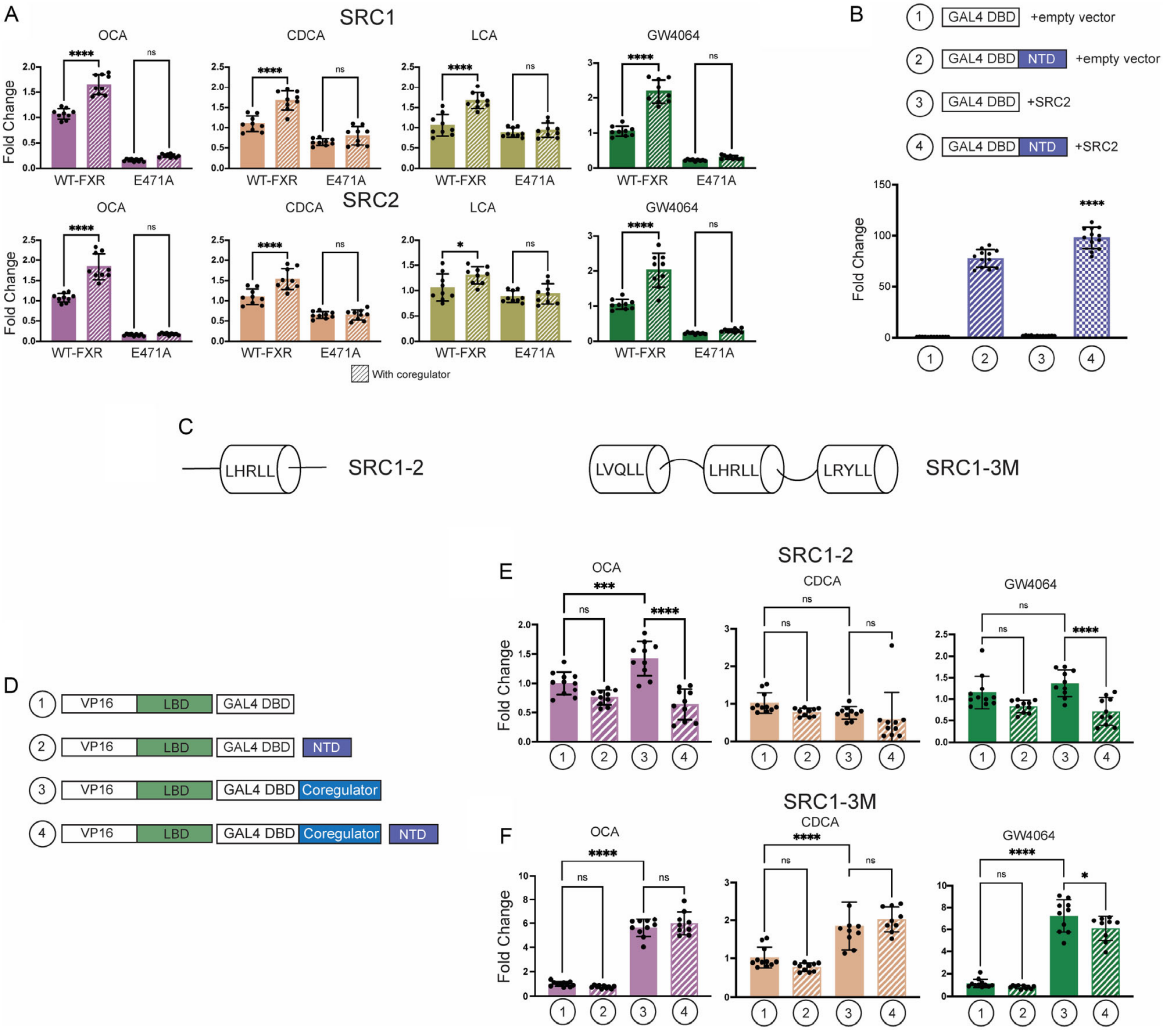
NTD interactions with coregulators. (A) Luciferase assays used to evaluate the effect of the E471A mutation on coregulator utilization with SRC1 and SRC2. (B) M1H assay using GAL4DBD‐NTD to assess the ligand‐independent activity of GAL4DBD‐NTD. (C) Schematic representation of the coregulator fragments used in the M2H system. (D) Schematic representation of the constructs used in the M2H assay. (E) Luciferase assays using the M2H system to evaluate the effect of the LBD on NTD and coregulator interaction. A decrease in fold change is observed in the one motif version when the NTD is added. (F) Luciferase assays using the M2H system to evaluate the effect of the NTD on the SRC1‐3M and LBD interaction. The addition of the NTD did not affect the LBD and SRC1‐3M interaction. Error bars for luciferase assay: standard deviation. One‐way and two‐way ANOVA, (****) *p* < 0.0001.

To understand how FXR interacts with transcriptional coregulators, we introduced an alanine mutation at the charge clamp residue: Glu471, located on H12 of the LBD. We evaluated E471A in a luciferase reporter using the IR1‐*luc* reporter construct. In WT‐FXR, co‐transfection of SRC1 and SRC2 potentiates the activity of FXR, confirming the interaction between FXR and the coregulators (Figure 2A). E471A revealed a drastic decrease in FXR activity, both in the presence and absence of coregulators. This result suggests that the FXR‐LBD plays a large role in coregulator interactions.

Despite the demonstrated interaction between FXR‐LBD and coregulators, several studies have shown interactions between NR NTDs and coregulator proteins [22, 23, 24, 25, 26]. To study the behavior of the FXR‐NTD independent of other domains, we first asked whether it can function as a transcriptional activator. We developed a mammalian one‐hybrid assay by fusing the NTD to Gal4DBD, allowing us to quantify the transcriptional activation of the isolated domain. We compared the activity of GAL4DBD‐FXR‐NTD to GAL4DBD alone, observing that the NTD alone induces a significant transcriptional response (Figure 2B). Co‐transfection of SRC2 coregulator further potentiates the activity (Figure 2B), supporting the interaction between FXR‐NTD and coregulators independent of other FXR domains, similar to those observed in other NRs. We note that this activity is also observed independently of RXR.

Next, we sought to investigate the behavior of the NTD in the context of the FXR‐LBD–coregulator interaction. We designed an M2H system to assay the LBD–coregulator interaction using VP16‐FXR‐LBD and GAL4DBD‐SRC1‐2 fusion constructs. The GAL4DBD‐SRC1‐2 construct contains the SRC1‐2 coregulator peptide (LTERHKILHRLLQEGSPSD) cloned next to the GAL4DBD (Figure 2C). To determine whether the NTD affects this interaction between the coregulator peptide and the LBD, we co‐transfected an NTD‐only construct (Figure 2D), generated by cloning the FXR‐NTD into pcDNA3.1 (see Section 4). We observed that the addition of the NTD results in a decreased fold change, suggesting that the disordered domain may interfere with LBD–coregulator interaction. This was observed with OCA‐ and GW4064‐treated cells, but not CDCA (Figure 2E).

We also created a multiple coregulator peptide construct to determine how the presence of multiple LXXLL motifs would affect NTD‐mediated disruption of the LBD–coregulator interaction. The construct GAL4DBD‐SRC1‐3M (SRC1‐3M) (Figure 2C) was generated by cloning the receptor‐interaction domain of SRC1 into the GAL4DBD plasmid. This fragment, containing three LXXLL motifs, interacts with the LBD of NRs, e.g., PPARγ [38]. Interestingly, when we used SRC1‐3M, no effect on the LBD–coregulator interaction was observed upon addition of the NTD (Figure 2F). Additionally, SRC1‐3M gives substantially higher transcription compared to SRC1‐2. This result strongly suggests that the presence of multiple coregulator motifs strengthens the LBD–coregulator interaction, preventing disruption by the NTD.

### 2.3 A Novel SXXLF Motif in the FXR‐NTD Mediates Interactions With the LBD

**FIGURE 3:**
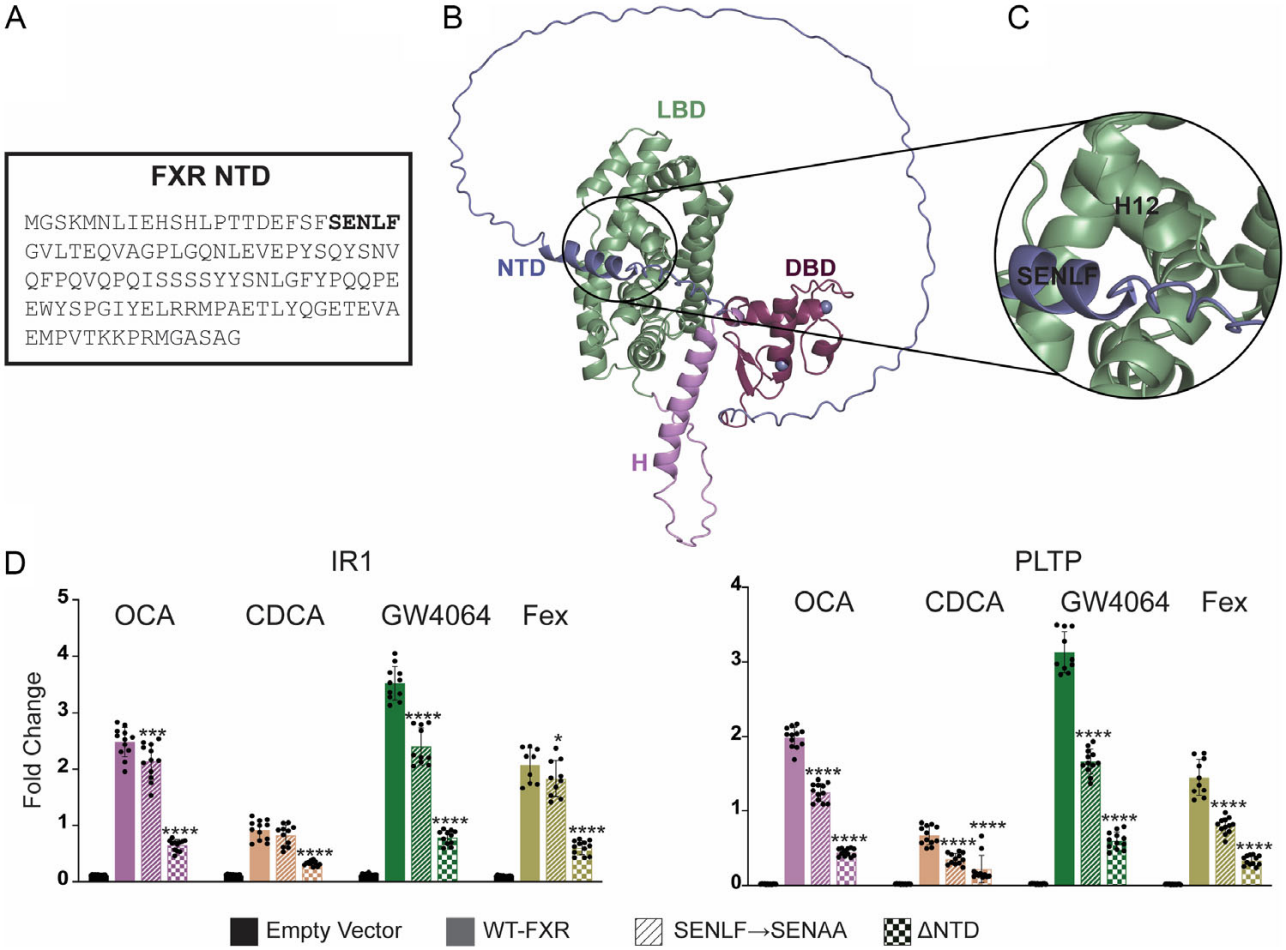
The SENLF motif mediates NTD transcriptional activity. (A) FXR‐NTD sequence with the SENLF motif bolded. (B) AlphaFold 3 model of full‐length FXR. (C) The SENLF motif from the NTD is positioned next to H12 in the LBD. (D) Luciferase assays evaluating the effect of the SENAA mutation on FXR activity using the IR1‐*luc* and PLTP‐*luc* constructs. After the mutations, a reduction in fold change was observed.

Due to the disordered nature of NR NTDs, no experimental structural studies have resolved the domain, precluding the ability to generate a structural understanding of how it interacts with other domains. Similar to what was observed in the AR, we hypothesized that motifs in the FXR‐NTD could mediate interdomain and coregulator interactions. We analyzed the NTD sequence in search of known motifs: LXXLL, WXXLF, or FXXLF. While none of these were found, the closest motif identified was SENLF (Figure 3A). In particular, SENLF is similar to FQNLF in the NTD of the AR, found to participate in agonist‐dependent LBD/AF‐2 interactions [28, 35, 36, 39, 40, 41]. To gain insight into the FXR‐NTD structure and the predicted secondary structure of these two motifs, we generated models of full‐length FXR, including the NTD. Models were generated using AlphaFold 3 and, as expected, revealed a highly unstructured FXR NTD (average confidence score: 24.23), with intact DBD and LBD (Figure 3B, Table S1A–D). Interestingly, the SENLF sequence (average confidence score: 29.48) forms a helix and docks near the AF‐2 surface of the LBD (Figure 3C, Table S1).

To determine whether this sequence is a valid recognition motif in the FXR‐NTD, we mutated it to SENAA in our pcDNA3.1‐FXR construct and tested the mutant receptor in the luciferase assay. With both reporter constructs (IR1‐*luc*, PLTP‐*luc*), the SENAA construct significantly decreases fold activation compared to WT‐FXR (Figure 3D). This is observed in all conditions, except for CDCA with the IR1‐*luc* element, validating the importance of the motif in FXR function. Interestingly, the activity levels of the SENAA mutant are not decreased to the same extent as ΔNTD, indicating that other parts of the NTD may facilitate the receptor's normal function.

**FIGURE 4:**
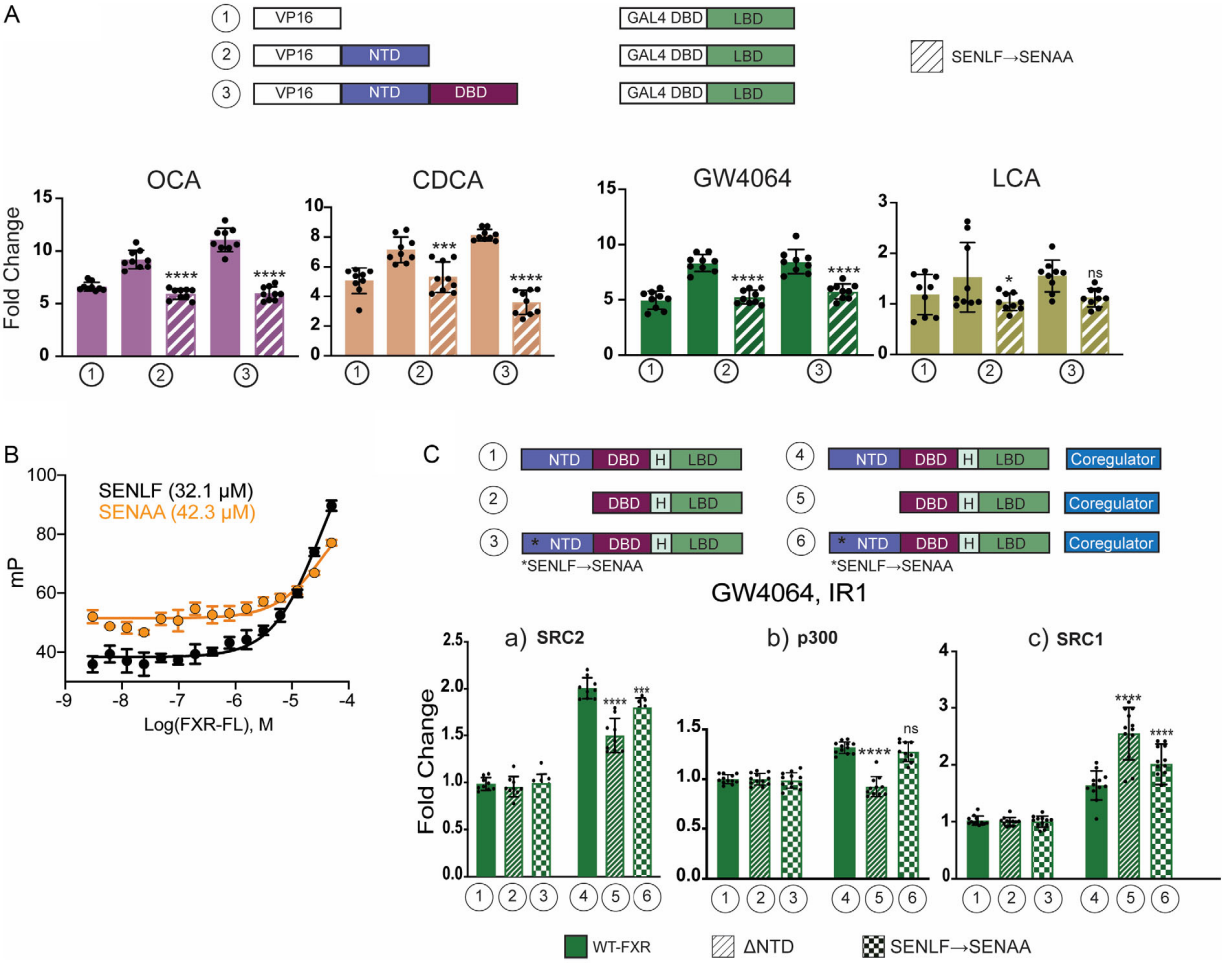
The SENLF motif binds FXR‐LBD and mediates coregulator interactions. (A) M2H assay to assess the effect of the SENAA mutation on the interdomain NTD–LBD contacts using the following constructs: NTD and NTD‐DBD in the VP16 vector with the LBD in the GAL4‐DBD vector. The SENAA mutation is associated with a reduction in interaction for conditions. (B) Fluorescence polarization–based binding assay using a fragment of the NTD containing the SENLF motif and the SENAA mutant. An EC_50_ of 32.1 µM was obtained for the SENLF peptide, and an EC_50_ of 42.3 µM was obtained for SENAA. (C) Luciferase assays evaluating the effect of the SENAA mutation on coregulator utilization, normalized to the constructs with no coregulator. Error bars for luciferase assay: standard deviation. (a) SRC2. (b) p300. (c) SRC1. (A) One‐way ANOVA and (C) two‐way ANOVA, (****) *p* < 0.0001.

To determine whether SENLF is an LBD recognition motif, we inserted the SENAA mutation into all M2H NTD constructs (NTD and NTD‐DBD) and tested their effect on the LBD–NTD interaction. Compared to WT constructs, the SENAA mutant decreases the fold change, suggesting that the NTD–LBD interaction is disrupted (Figure 4A). The decrease was observed in cells treated with OCA, CDCA, and GW4064. We also used a fluorescence polarization peptide‐binding assay to assess whether this motif affects the NTD–LBD interaction. We synthesized a fluorescein‐tagged 20‐amino acid fragment from the NTD containing the SENLF motif (PTTDEFSFSENLFGVLTEQV), along with the SENAA variant (PTTDEFSFSENAAGVLTEQV), and measured binding to purified, full‐length FXR (DBD‐hinge‐LBD). An EC_50_ of 32.1 µM is calculated for the SENLF peptide, indicating weak binding. A modest reduction in EC_50_ (42.3 µM) is observed for the SENAA peptide, supporting the relevance of the motif in NTD–LBD binding (Figure 4B).

### 2.4 The SXXLF Motif Selectively Mediates NTD–Coregulator Interactions

Next, we asked whether the SENLF motif plays a role in coregulator recognition. To enable a thorough comparison, we tested WT‐FXR, ΔNTD, and SENAA, both in the absence (Figure 4C, Conditions 1–3) and presence of coregulators (Conditions 4–6), to determine how added coregulators influence FXR‐driven transcription. Our selection of coregulators included SRC1, SRC2, and p300. To quantify the effect of the mutations on coregulator activity, the data are normalized to the corresponding no‐coregulator control for each group (i.e., Conditions 4, 5, and 6 are normalized to 1, 2, and 3 respectively). When co‐transfected with WT‐FXR, all three coregulators increased the fold change, confirming their interaction with FXR (Figure 4C, Conditions 1 vs. 4). As the ΔNTD mutant should completely abolish NTD–coregulator interactions, we hypothesized that ΔNTD + coregulator would have lower transcriptional activity, compared to WT‐FXR + coregulator. This was observed in SRC2 and p300, confirming that NTD interacts productively with these coregulators. Unexpectedly, we observe higher transcriptional activity with SRC1 in the ΔNTD compared to WT‐FXR. This observation may suggest that the NTD interferes with a productive interaction between FXR‐LBD and SRC1, and its removal allows more effective coregulator usage.

The SENAA mutant shows varying behaviors among the three coregulators. With SRC2, a small but statistically significant reduction in activity is observed compared to WT‐FXR, suggesting a potentially modest role of the SENLF motif in SRC2 interaction. However, this activity is higher than ΔNTD, which suggests other motifs in the NTD may also interact with SRC2 (Figure 4C (a), Condition 6). We do not observe a significant difference between WT‐FXR and the SENAA mutant interactions with p300, suggesting that this motif may not actively recruit p300 (Figure 4C (b), Condition 6). Finally, the SENAA mutant shows higher activity with SRC1 (Figure 4C (c), Condition 6) compared to WT‐FXR but lower than that of ΔNTD (Figure 4C (c), Condition 5), further supporting the likely interpretation that the NTD interferes with the interaction between FXR and SRC1. The observation also lends further support to the existence of other interaction motifs in the NTD. Additionally, we note that in our assay, ΔNTD interacting with p300 (Figure 4C (b), Condition 5) gives a similar fold change to the no‐coregulator control, which is not observed in the SRC coregulators. This result suggests that FXR interactions with p300 may be mediated entirely by the NTD. Collectively, these data demonstrate that FXR interacts with coregulators with varying mechanisms, including mixed usage of the LBD and NTD.

### 2.5 SXXLF Mutation Influences Interdomain Contact and Coupling in Simulations

**FIGURE 5:**
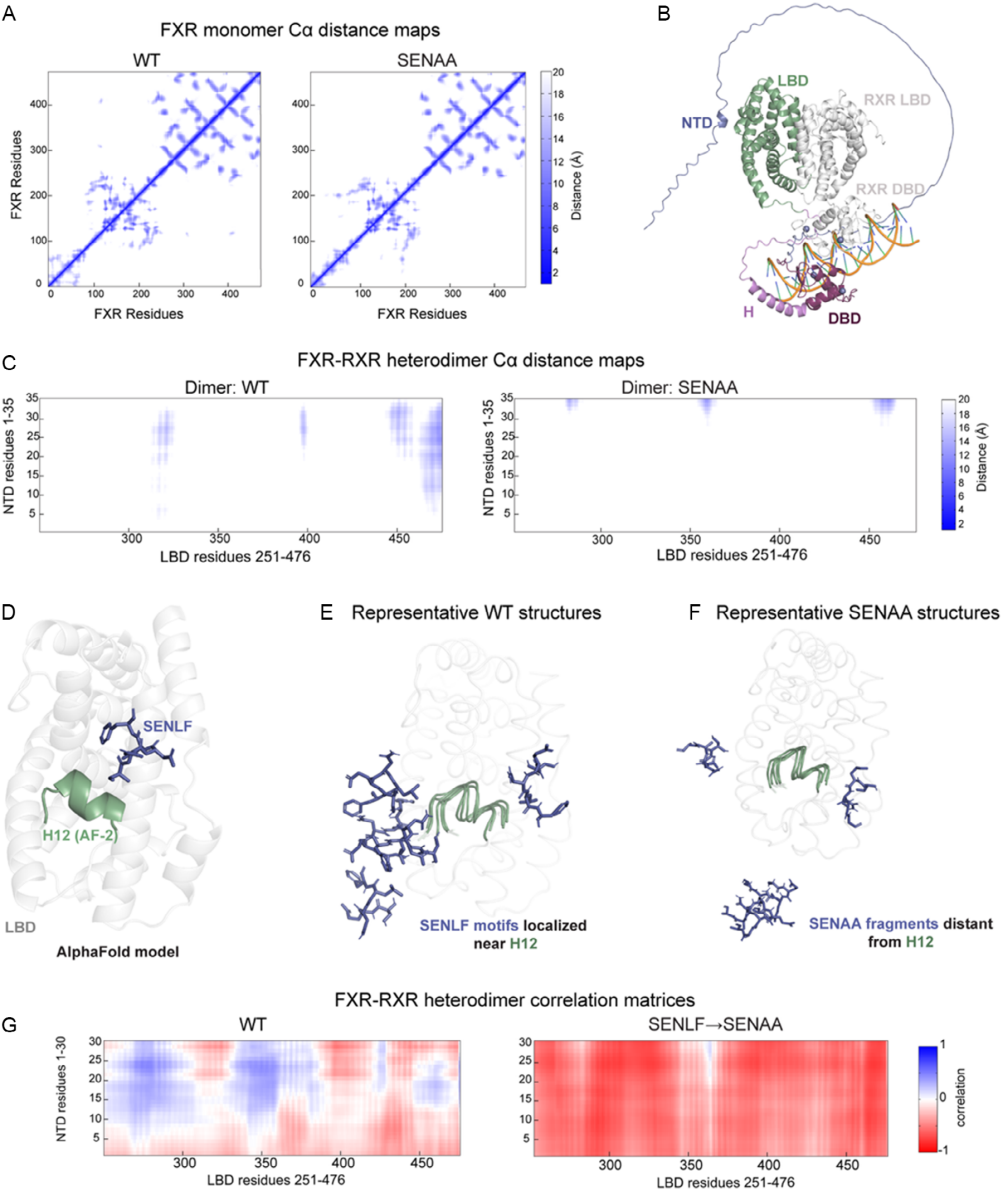
Effect of SENAA mutant on FXR dynamics. (A) C‐alpha distance maps comparing contacts in monomeric WT and SENAA FXR mutant. (B) AlphaFold 3 model of the FXR‐RXR heterodimer, with FXR‐NTD included. (C) C‐alpha distance maps comparing contacts in WT and SENAA FXR‐RXR heterodimers. (D) AlphaFold 3 model of the isolated LBD showing the position of the SENLF residues to H12. (E) Clustering representation of WT‐FXR showcasing the SENLF residues positioned near H12. (F) Clustering representation of SENAA FXR mutant with the SENAA residues positioned distally from H12. (G) Correlation matrices comparing differences in NTD–LBD correlation between WT and SENAA FXR‐RXR heterodimers.

To investigate the effect of the SENAA mutation on FXR dynamics, we performed MD simulations of both WT and SENAA FXR models. Six replica 5‐μs trajectories were obtained for each monomeric model and combined prior to analysis. A contact analysis was performed to identify differences in contacts between both complexes. Only a handful of minor differences were observed between the two contact maps (Figure 5A), indicating that no major conformational changes are predicted between WT and mutant monomeric FXR. We hypothesized that conformational effects might be observed in a more physiologically relevant context. Thus, we constructed a model of the DNA‐bound FXR‐RXR heterodimer, where FXR contains all domains, including NTD, while RXR only contains DBD, hinge, and LBD (Figure 5B). The RXR‐NTD was omitted to remove any confounding effects of a second large, disordered region. The IR1 consensus DNA sequence, which represents the canonical FXR‐RXR response element, was used in the model. We generated the SENAA mutant of the heterodimer as well and ran MD simulations (four replicas of length 1.5–3.5 μs for each complex). The replicate simulations were combined prior to analysis. Contact maps reveal clear differences in the FXR‐NTD region of both complexes (Figure 5C). Extensive contacts are observed between amino acids 1–30 of the NTD (containing SENLF) and the C‐terminal region of the LBD (containing H12) in the WT complex. These contacts are absent in the SENAA mutant.

To visualize the major conformations present in the simulations, we performed clustering of the trajectories. Alignment to the LBD was performed, after which five clusters were obtained for both the combined WT and combined SENAA mutant dimer trajectories. As the AlphaFold model predicted the SENLF motif as being positioned next to H12 (Figure 5D), we focused on visualizing the position of the SENLF and SENAA segments relative to the LBD. In the WT‐FXR trajectories, the SENLF motif varied in position but always remained within a ~20 angstrom radius of H12 (Figure 5E). In contrast, the SENAA segment in the mutant trajectories was observed to be as far as ~40 angstroms in multiple conformations (Figure 5F). This observation shows that the SENAA mutation induces a conformational rearrangement in the FXR‐RXR heterodimer.

To further illustrate the conformational change induced by the in silico mutation, we identified two amino acid pairs: G27‐Q313 (NTD‐H4) and V43‐F282 (NTD‐H3) whose average Cα–Cα distances change significantly (*p* < 0.05, student's *t*‐test) between the WT and mutant complexes. The average G27‐Q313 distance changes from 18.7 Å in WT‐FXR to 37.2 Å in the SENAA mutant (Figure S2A, top). In the WT complex, the NTD is positioned proximally to H4, with an average distance of <20 Å. As H4 is part of AF‐2, this positioning reflects a close interaction between the NTD and AF‐2 regions. Specific NTD residues involved in this conformation are residues 28–33, which lie adjacent to the SENLF motif (22–26). In contrast, in the SENAA mutant, residues 28–33 are more distant from the H4, with an average distance of >35 Å (Figure S2B). This change in distances is summarized in a histogram (Figure S2C). In contrast, the distance between V43‐F282 decreases from 40.8 Å in FXR to 19.8 Å in the mutant (Figure S2A, bottom). The change reflects a shift in distance between the NTD (residues 35–48) and H3 of the LBD (Figure S2D), going from ~34 Å in WT‐FXR to under 20 Å in the SENAA mutant (summarized in Figure S2E). These large shifts in the position of the NTD highlight a significant conformational rearrangement that occurs in response to the mutation.

To characterize how the SENAA mutation potentially influences allosteric coupling between the NTD and LBD, we measured the correlation between amino acids 1–30 of the NTD, which contain the SENLF motif in the NTD and the LBD (Figure 5G). A striking difference is observed in correlation patterns. While residues 1–30 of the SENAA mutant complex are anticorrelated with the entire LBD, several regions of positive correlation are observed in the WT complex. This includes the region beginning from H10 up to H12. This result suggests that the two amino acid mutations in the NTD dramatically alter conformation as well as interdomain coupling.

## 3 Discussion

NR NTDs are understudied, largely due to their intrinsically disordered nature. This work presents the first functional investigation of the FXR‐NTD. To define the roles of the FXR‐NTD, we first asked whether it interacts with other domains. Our data confirms these interdomain interactions. This result is not unexpected, based on precedents in the literature. We observe that adding the DBD enhances interdomain interactions. While the reason for this enhancement is not obvious from our experiments, previous reports on the PR indicate that the NTD undergoes structural stabilization when attached to the DBD, also playing roles in DNA binding [42]. The presence of additional FXR domains may impart increased structure to the disordered NTD, enhancing its interactions with other domains or binding partners. Biophysical experiments such as CD or NMR will be used in future experiments to test the hypothesis that additional domains increase the secondary structure of the NTD.

We show that the FXR‐NTD can interact with coregulators, as determined by our M2H assay. When a coregulator construct containing one SRC1 LXXLL motif was transfected with the LBD and NTD, we observed a reduction in the interaction between LBD and the coregulator, suggesting a competition between the NTD and LBD for coregulator interaction. Use of the SRC1‐3M construct allowed interaction with LBD to be observed, even with co‐transfection of the NTD. This result strongly supports an enhanced interaction between the SRC1‐3M motif and the FXR‐LBD, which resists interference from the NTD. Our mammalian one‐hybrid (M1H) assay also revealed ligand‐independent transcriptional activity in the NTD alone, providing further support that it can interact with coregulators [43, 44, 45]. We note that all of our coregulator interaction studies were performed in the absence of RXR, the heterodimer partner for FXR. Because RXR LBD and potentially the RXR‐NTD can interact with coregulators, excluding this receptor was necessary to isolate specific interactions involving only FXR NTD. Limitations of this approach are acknowledged, e.g., in the luciferase‐based assays, where higher fold changes would be observed with co‐transfection of RXR. We have validated the SENLF motif as a coregulator interaction motif. SXXLF represents a novel recognition motif, with WXXLF and FXXLF from AR being the first time that noncanonical LXXLL coregulator motifs were identified in a NR [27, 46]. We have now expanded this and have established that SENLF is another recognition sequence for FXR. Noncanonical LXXLL motifs were identified in the orphan NR DAX‐1, which are involved in repressing the activity of other receptors SF‐1 and LRH1 [47]. Unveiling this new motif in FXR allows us to probe more deeply regarding how FXR selectively recruits certain coregulators and/or binding partners. For instance, do different motifs preferentially interact with specific coregulators? Our findings also suggest that the full set of NR recognition motifs is not yet known. The identification of SXXLF and related motifs in NRs and other proteins may drive the discovery of novel NR‐interacting partners.

But interestingly, SENLF alone does not capture the full functional contribution of the NTD. We observed this because the SENAA mutant did not reduce FXR transcriptional activity to the same extent as the ΔNTD mutant (Figure 3D). This finding strongly implicates other regions of the NTD in coregulator interactions and/or crucial NR functions. Future work to identify additional motifs in the NTDs (e.g., by segment deletions, alanine scanning, etc.), as well as elucidate the domain's mechanism of action, will be required [48].

Importantly, our work focuses on the FXRα1 isoform, but we note that the NTDs of isoforms FXRα3 and FXRα4 have an extended NTD, compared to those of FXRα1 and FXRα2 [7]. Interestingly, the extended NTD sequence does not contain the SENLF motif, which indicates that other isoforms utilize distinct mechanisms for these functions.

In summary, our work confirms two roles for the FXR‐NTD: interactions with the LBD and recognition of coregulators. This work highlights the complexity of NR–coregulator interactions, as we observed that the ΔNTD but not the SENAA mutation impaired recruitment of the p300 coregulator. Our findings also hint at coregulator specificity of the SENLF motif, suggesting that it may be important for certain coregulators but not others. We also observed varying effects of the SENAA mutation vs. ΔNTD on the transcriptional interaction with SRC1 and SRC2, suggesting that coregulators may have a stronger interaction with either the FXR‐LBD or NTD. The underlying cause for this phenomenon will be explored in future studies. While the LBD appears to be the most essential factor for binding coregulators, it remains possible that some coregulators prefer the NTD. Our future studies will test a larger swarth of coregulators to understand their partitioning between the two domains.

## 4 Methods

### 4.1 List of Primers

**Table:** 

Primers	Sequence
FXR PCDNA3.1_FWD	5′ GCTTGGTACCATGGGATCAAAAATGAATCTCATT 3′
FXR PCDNA3.1_REV	5′ AACCCTCGAGCTGCACGTCCCAGATTTC 3′
NTD_25‐26AA_FWD	5′ TTTAACAGAACAAGTGGCAGGTCCTCTGGGA 3′
NTD_25‐26AA_REV	5′ AAAAAGAAAATTCATCTGTGGTAGGTAAATGGGA 3′
NTD_25‐26A INS	5′ ACCACAGATGAATTTTCTTTTTCTGAAAATGCCGCCGGTGTTTTAACAGAACAAGTGGCA 3′
No_NTD INS	5′ TTATCGAAATTAATACGACTCACTATAGGGAGACCCAAGCTGGCTAGCGTTTAAACTTAAGCTTGGTACCAGGATCAAAG 3′
minP_FWD	5′ AGACACTAGAGGGTATATAATGGAAGCTCGACTTCCAG CTTGGCAATCCGGTACTGT 3′
pgl4_REV	5′ CGGTACCGGCCAGTTAGGCC 3′
IR1_INS	5′ AACTGGCCGGTACCGTCAAG**AGGTCATTGACCT**TTTTGTCAAG**AGGTCATTGACCT**TTTTGAGACACTAGAGGGT 3′
PLTP_INS	5′ AACTGGCCGGTACCGCAACTGA**GGGTCAGTGACCC**AAGTGACAACTGA**GGGTCAGTGACCC**AAGTGAAGACACTAGAGGGTA 3′
3M BIND_FWD	5′ TAGATAAAGATGAGAAAGATTGAATAACTAAGGCCGCTTCCCT 3′
3M BIND_REV	5′ TATTTACTGTCTCCATCTGACGATACAGTCAACTGTCTTTGACCTTT 3′
3M INS_FWD	5′ AAAGACAGTTGACTGTATCGTCAGATGGAGACAGTAAATACTCTCAAACCAGT 3′
3M INS_REV	5′ GAAGCGGCCTTAGTTATTCAATCTTTCTCATCTTTATCTAAAAGATAGCGTAGGAG 3′

### 4.2 Cloning/Constructs

Human FXRα (NR1H4, UniProt Q96RI1‐1) was cloned into the pcDNA3.1(+) expression vector using forward and reverse primers that contained the restriction sites for EcoRI and HindIII. The ΔNTD was made by using PCR to amplify the region of FXR after the NTD and then using this insert (ΔNTD‐INS) to assemble the plasmid with a start codon added to the DBD. The constructs for the luciferase reporter assays were made by modifying pGL4.31(luc2P/GAL4UAS/Hygro) with two copies of the FXRE and the minimal promoter upstream of the firefly luciferase gene. pGL4.31 was first digested by Dpn1‐HF and HindIII‐HF and PCR amplified with primers (minP_FWD and pgl4_REV) to append the minimal promoter. Then, two copies of the FXRE sequence IR1 and PLTP were inserted using Gibson assembly.

GenScript mainly synthesized the constructs used in the mammalian two‐hybrid (M2H) protein–protein interaction assay. The LBD of FXR (residues 245–476) was inserted after the GAL4‐DBD in the pBIND vector (Promega) to give GAL4‐FXR‐LBD, and it was also inserted downstream of the VP16 gene in the pACT vector (Promega) to give VP16‐LBD. For the NTD (residues 1–119) and NTD‐DBD (residues 1–196) constructs, they were all individually cloned into the pACT vector downstream of the VP16 gene to give rise to VP16‐NTD, VP16‐NTD‐DBD, and VP16‐NTD‐DBD‐H. For GAL4‐NTD, the NTD (residues 1–119) was inserted after the GAL4‐DBD. For the coregulators used in the hybrid assay, the NR recognition motif of SRC1 (residues: LTERHKILHRLLQEGSPSD) was inserted at the N‐terminus of the GAL4DBD, yielding GAL4‐SRC1‐2. The GAL4‐SRC1‐3M construct was generated by cloning residues 620–759 (Human NCOA1, SRC1α, Uniprot Q15788‐1) into the pBIND vector downstream of the GAL4 gene using Gibson assembly.

### 4.3 Luciferase Assays

#### 4.3.1 Cell Lines and Cell Culture

Cell reporter assays were performed with HeLa cells (American Type Culture Collection) grown in Dulbecco's Modified Eagle Medium (Thermo Fisher Scientific) with 10% fetal bovine serum and 1% L‐glutamine and incubated at 37°C in a humidified atmosphere of 5% CO_2_.

#### 4.3.2 Transfection and Dual‐Luciferase Assay

Luciferase assays and transfections were performed as we have previously described [49]. HeLa cells were seeded at 10,000 cells/well in 96‐well, clear flat‐bottom cell culture plates. Cotransfection was performed with 5 ng of the expression plasmid pcDNA3.1‐FXR, 50 ng of the pGL4.31‐derived plasmids containing the luciferase gene downstream of the FXRE, and 1 ng of renilla luciferase (pRL‐CMV). The FXREs used were IR1 (inverted repeat spaced by one nucleotide) sequences, the IR1 consensus sequence (AGGTCAtTGACCT), and the PLTP IR1 sequence. The FXRE plasmids used in this assay contained two copies of their respective response element sequences. Transfection was repeated at least in triplicate with Fugene HD (Promega). In assays with added whole coregulator or mutated coregulator, 10 ng of plasmids containing pSG5‐SRC1, pcDNA3.1‐SRC2, and pDEST26‐p300 was used (coregulator plasmids from Dr. Eric Ortlund (Emory University)).

Twenty‐four hours after transfection, cells were treated with DMSO (final concentration of 0.37%) or with test ligands to a final concentration of 10 or 100 µM. After 24‐h incubation, firefly and renilla luciferase activity was assayed using the Dual Glo kit (Promega) on a SpectraMax iD5 plate reader. Data is represented as a fold change, which is the firefly signal over the renilla signal, normalized to the DMSO‐treated empty vector controls unless otherwise specified in the figure caption.

Data from the luciferase assays are presented here as the mean ± standard deviation (SD) of three biological replicates. *p*‐values were calculated using two‐way ANOVA with Dunnett's multiple comparisons test to evaluate statistical significance between mean comparisons. One‐way ANOVA was also used for data when comparing means across multiple groups while focusing on a single factor. Significance levels are as follows: *p* > 0.05 (ns), *p* < 0.05 (*), *p* < 0.01 (**), *p* < 0.001 (***), *p* < 0.0001 (****). Analysis was performed using GraphPad Prism (v. 10.6.0).

#### 4.3.3 Mammalian Two‐Hybrid Assay

For this assay, the instructions from the CheckMate M2H System (Promega) were used. Transfection was performed using equal amounts (5 ng) of each pBIND and pACT construct, along with 50 ng of the reporter plasmid pG5luc, which contains the firefly luciferase gene downstream of the activating response element for the GAL4‐DBD. Controls were included in wells with empty pACT or pBIND vectors. Treatment of cells, measurement of luciferase activity, and data processing were performed as described in the previous section.

#### 4.3.4 Mammalian One‐Hybrid Assay

For this assay, transfection was performed using 5 ng of the pBIND constructs (GAL4‐NTD or GAL4‐empty) with 10 ng of SRC2 (when being used) and 50 ng of the reporter plasmid pG5luc. The control used the empty pBIND vector. Treatment of cells, measurement of luciferase activity, and data processing were performed as described in the previous section.

### 4.4 Fluorescence Polarization Peptide Binding Assay

FXR residues 120–476 (DBD‐hinge‐LBD) were expressed and purified as we have previously described [6]. For the binding assay, fluorescein (FAM)‐labeled peptides consisting of the SENLF motif, along with residues surrounding the motif, Pep1: PTTDEFSFSENLFGVLTEQV, and the mutant form Pep2: PTTDEFSFSENAAGVLTEQV, were synthesized by LifeTein, USA. The peptides were diluted 2× in the assay buffer (50 mM HEPES, pH 7.4, 150 mM NaCl, 5% glycerol, 1 mM DTT, and 20 µM CDCA). The peptides were added to a final concentration of 400 nM to serially diluted FXR (50 µM to 3 nM) in a 384‐well black‐bottom plate. The protein contains the following substitutions: C77S, C88S, C124S, C317S, and C351S, which allow for a higher protein concentration without aggregation. The plate was centrifuged at 1000 rpm for 1 min and incubated at room temperature for 2 h before reading. Polarization was monitored on a SpectraMax iD5 plate reader (Molecular Devices, USA) with excitation and emission wavelengths of 485/528. Each experiment was conducted as three independent replicates with the buffer background subtracted before plotting. The data were compiled from all replicates and plotted as protein concentration versus the change in millipolarization. Binding saturation data was fit to a dose–response (three parameters) equation in GraphPad Prism (v 10.6.0).

### 4.5 MD Simulations

FXR models for monomeric (NTD + DBD + H+LBD + DNA) and heterodimer (FXR NTD + DBD + H+LBD and RXR DBD + H + LBD + DNA) forms of the receptor were generated using AlphaFold 3 [50]. For the monomeric versions of WT and SENAA FXR, six 5‐µs replicate simulations were carried out. The protein models were parameterized using the tLEaP program using the ff14SB protein force field [51]. The complexes were then solvated in an octahedral TIP3P water box with a 10 Å buffer and a sodium chloride salt concentration of 150 mM to mimic physiological conditions [51]. Simulations were minimized, heated, equilibrated, and run with Amber18/20/22 [52, 53]. Simulation equilibration and production were achieved with GPU acceleration [54]. Four minimization steps were completed, each consisting of 5000 steepest descent steps followed by 5000 conjugate gradient steps. The minimization steps are as follows: (1) 500 kcal/mol Å^2^ restraints on solute atoms; (2) 100 kcal/mol Å^2^ restraints on solute atoms; (3) 100 kcal/mol Å^2^ restraints on the ligand, Zn^2+^ ions, and DNA present; and (4) no restraints on the system. In these instances, solute does not refer to sodium chloride ions in solution. After minimization, the system was heated with a 100 ps *NVT* simulation with all atoms under 5 kcal/mol Å^2^ of restraints. Then, pre‐equilibration was completed in three 10 ns steps: (1) 10 kcal/mol Å^2^ restraints on solute atoms; (2) 1 kcal/mol Å^2^ restraints on solute atoms; and (3) 1 kcal/mol Å^2^ restraints on the ligand, Zn^2+^ atoms, and DNA present. All simulations used SHAKE with a 2 fs time step.

The simulations for the heterodimer WT and SENAA FXR complexes were run on the Anton 3 [55] supercomputer. The complexes were solvated in a cubic box with a NaCl buffer of varying size, ensuring a total number of atoms exceeding 100,000. Heating, minimization, and pre‐equilibration were performed as described above. Four replicas of lengths 1.5–3.5 µs were generated for each complex and combined prior to analysis. The heating, minimization, and equilibration for classical MD simulation follow the protocol previously published by our group. Cα distance maps and correlation matrices were generated using CPPTRAJ [56, 57].

## Funding

This study was supported by the Directorate for Biological Sciences (CAREER: 2144679) and the National Institute of General Medical Sciences (DP2‐GM149753, T32GM149417, and T32GM152354).

## Conflicts of Interest

The authors declare no conflicts of interest.

## Supporting information

Supplementary Material
