# Supplementary material for: A Novel SXXLF Motif in the FXR N‐Terminal Domain Mediates Coregulator and Interdomain Interactions
Source: Chembiochem. 2026 Sep 10;27(17):e202500967. doi: 10.1002/cbic.202500967 (PMC13562826; doi:10.1002/cbic.202500967)
Supplement: Supplementary file 1 [file CBIC-27-e202500967-s001.pdf]

Supporting Information

A novel SXXLF motif in the FXR N-terminal domain mediates coregulator and interdomain interactions

Priscilla Villalona[1*], Thilini Pulahinge[2*], Tracy Yu[1], Jordan Wenning [1], Sabab Hasan Khan[1], Crawford Joseph Frisbie[1], Jill Magafas[1], Addison Barbe[1], C. Denise Okafor [1,2‡]

[1]Department of Biochemistry and Molecular Biology, The Pennsylvania State University, University Park, PA 16802

[2]Department of Chemistry, The Pennsylvania State University, University Park, PA 16802

*Authors contributed equally

‡cdo5093@psu.edu

A) FXR-NTD

Residue_ID	pLDDT	Residue_ID	pLDDT	Residue_ID	pLDDT	Residue_ID	pLDDT	Residue_ID	pLDDT	Residue_ID	pLDDT
1	28.55	21	28.14	41	21.49	61	21.52	81	21.29	101	23.34
2	23.02	22	27.89	42	24.99	62	25.91	82	22.99	102	25.81
3	24.73	23	26.36	43	18.82	63	24.37	83	20.5	103	21.47
4	22.76	24	30.71	44	21.3	64	27.89	84	20.66	104	29.53
5	19.86	25	31.73	45	21.67	65	28.92	85	19.68	105	25.91
6	19.93	26	31.12	46	20.61	66	26.6	86	19.67	106	24.61
7	20.58	27	34.4	47	21.95	67	28.8	87	18.3	107	32.09
8	22.76	28	27.98	48	19.12	68	29.32	88	17.38	108	25.06
9	22.69	29	25.61	49	19.14	69	26.5	89	19.3	109	32.78
10	23.6	30	28.36	50	23.61	70	22.71	90	18.79	110	34.62
11	28	31	24.52	51	24.79	71	25.14	91	20.84	111	29.59
12	22.68	32	23.78	52	21.93	72	18.37	92	23.21	112	31.12
13	22.34	33	23.19	53	24.29	73	19.81	93	22.4	113	35.09
14	25.64	34	26.63	54	19.03	74	19.68	94	20.33	114	24.78
15	22.05	35	31.09	55	24.18	75	20.57	95	21.79	115	30.73
16	23.86	36	28.16	56	24.58	76	19.96	96	19.75	116	26.97
17	21.93	37	21.47	57	20.48	77	23.19	97	21.41	117	29.21
18	21.53	38	27.43	58	23.5	78	22.15	98	22.54	118	30.16
19	21.52	39	23.53	59	22.9	79	19.69	99	22.03	119	31.13
20	25.21	40	24.93	60	22.83	80	19.93	100	24.54		

B) FXR-DBD

Residue_ID	pLDDT	Residue_ID	pLDDT	Residue_ID	pLDDT	Residue_ID	pLDDT
120	33.64	140	81.09	160	93.07	180	93.84
121	39.51	141	83.24	161	85.49	181	93.18
122	38.99	142	85.56	162	92.78	182	93.7
123	58.56	143	90.01	163	90.03	183	95.46
124	76.4	144	91.06	164	92.91	184	92.55
125	81.61	145	91.72	165	91.03	185	90.93
126	87.32	146	92.77	166	92.31	186	90.41
127	87.72	147	93.31	167	91.51	187	89.3
128	91.58	148	92.12	168	93.03	188	84.45
129	91.5	149	92.39	169	91.64	189	85.85
130	92.33	150	90.34	170	93.36	190	83.73
131	91.65	151	89.5	171	90.33	191	79.7
132	93.73	152	85.88	172	93.49	192	85.51
133	91.12	153	87.57	173	91.97	193	81.46
134	88.75	154	88.4	174	90.57	194	79.19
135	86.89	155	85.69	175	92	195	71.04
136	82.99	156	81.93	176	94.19	196	70.19
137	80.4	157	85.38	177	92.13		
138	82.85	158	88.64	178	92.22		
139	80.93	159	88.85	179	93.42		

C) FXR-Hinge

Residue_ID	pLDDT	Residue_ID	pLDDT	Residue_ID	pLDDT
197	67.71	217	55.3	237	33.26
198	59.52	218	61.57	238	27.83
199	57.53	219	53.29	239	34.73
200	57.27	220	53.19	240	33.79
201	54.05	221	54.5	241	36.77
202	53.43	222	55.64	242	39.71
203	56.45	223	52.57	243	44.63
204	60.38	224	45.75	244	51.82
205	64.4	225	40.28		
206	58.89	226	35.83		
207	59.83	227	40.34		
208	63.19	228	36.68		
209	60.75	229	34.71		
210	57.42	230	37.05		
211	60.16	231	32.37		
212	62.37	232	35.89		
213	57.42	233	29.52		
214	61.1	234	32.47		
215	61.53	235	31.45		
216	58.73	236	35.91		

D) FXR-LBD

Residue_ID	pLDDT	Residue_ID	pLDDT	Residue_ID	pLDDT	Residue_ID	pLDDT	Residue_ID	pLDDT	Residue_ID	pLDDT
245	58.82	285	82.54	325	94.64	365	86.44	405	97.28	445	92.12
246	65.43	286	83.91	326	94.38	366	89.52	406	96.59	446	90.71
247	79.77	287	85.21	327	95.63	367	90.3	407	97.32	447	90.58
248	91.86	288	84.71	328	94.61	368	92.06	408	97.78	448	89.44
249	95.14	289	85.42	329	94.97	369	92.46	409	96.93	449	88.45
250	96.19	290	86.98	330	95.58	370	92.06	410	96.97	450	86.52
251	96.51	291	87.27	331	95.51	371	92.2	411	96.97	451	87.97
252	96.03	292	88.89	332	93.12	372	94.07	412	97.47	452	88.35
253	96.98	293	88.58	333	94.4	373	93.58	413	96.19	453	85.43
254	96.58	294	88.98	334	95.25	374	92.06	414	96.85	454	84.78
255	95.62	295	90.13	335	93.08	375	93.39	415	97.45	455	83.66
256	96.31	296	91.69	336	90.75	376	95.49	416	96.91	456	81.5
257	97.43	297	90.04	337	93.95	377	93.93	417	96.4	457	80.18
258	96.49	298	90.55	338	92.54	378	93.92	418	97.62	458	76.8
259	95.01	299	91.8	339	88.64	379	95.09	419	97.71	459	73.42
260	96.66	300	91.48	340	88.26	380	94.39	420	96.68	460	69.08
261	96.62	301	91.34	341	87.82	381	95.51	421	96.7	461	67.95
262	94.7	302	92.8	342	79.17	382	95.41	422	96.44	462	68.25
263	93.35	303	93.29	343	66.94	383	95.65	423	96.25	463	70.54
264	94.94	304	93.35	344	68.39	384	96.61	424	95.47	464	80.89
265	91.63	305	95.38	345	73.4	385	97.07	425	94.68	465	84.43
266	88.2	306	95.86	346	71.77	386	96.83	426	92.61	466	87.27
267	77.58	307	94.43	347	74.37	387	97.46	427	93.64	467	87.07
268	81.43	308	93.44	348	79.09	388	97.94	428	93.56	468	88.46
269	78.44	309	95.38	349	81.91	389	97.63	429	93.2	469	88.85
270	83.26	310	94.13	350	83.14	390	97.5	430	93.29	470	87.07
271	81.68	311	95.18	351	84.04	391	97.81	431	90.69	471	86.55
272	83.91	312	96.8	352	85.09	392	97.69	432	92.64	472	88.26
273	83.41	313	96.2	353	86.04	393	97.53	433	94.73	473	88.07
274	81.49	314	96.01	354	85.89	394	97.63	434	95.45	474	82.95
275	82.17	315	96.71	355	85.72	395	97.78	435	93.93	475	80.33
276	83.32	316	96.67	356	86.06	396	96.88	436	92.83	476	73.36
277	81.83	317	96.28	357	87.71	397	96.26	437	96.23		
278	79.81	318	96.14	358	86.42	398	93.54	438	94.83		
279	78.98	319	97.49	359	83.51	399	94.49	439	93.05		
280	68.56	320	96.93	360	81.57	400	92.13	440	93.54		
281	74.04	321	95.59	361	83.27	401	94.11	441	92.54		
282	75.64	322	96.37	362	86	402	96.44	442	90.19		
283	83.55	323	97.24	363	87.83	403	96.8	443	91.46		
284	82.97	324	95.55	364	86.78	404	97.48	444	92.95		

Table S1. AlphaFold 3 confidence scores for monomeric FXR model. A, Confidence scores for FXR-NTD (residues 1-119). B, Confidence scores for FXR-DBD (residues 120-196). C, Confidence scores for hinge region (residues 197-244). D, Confidence scores for FXR-LBD (residues 245-476). Colors correspond to confidence scores with low scores (under 50) colored in orange, scores 50-69 are colored in yellow, scores 70-89 are in light blue, and scores 90 and above are in dark blue.

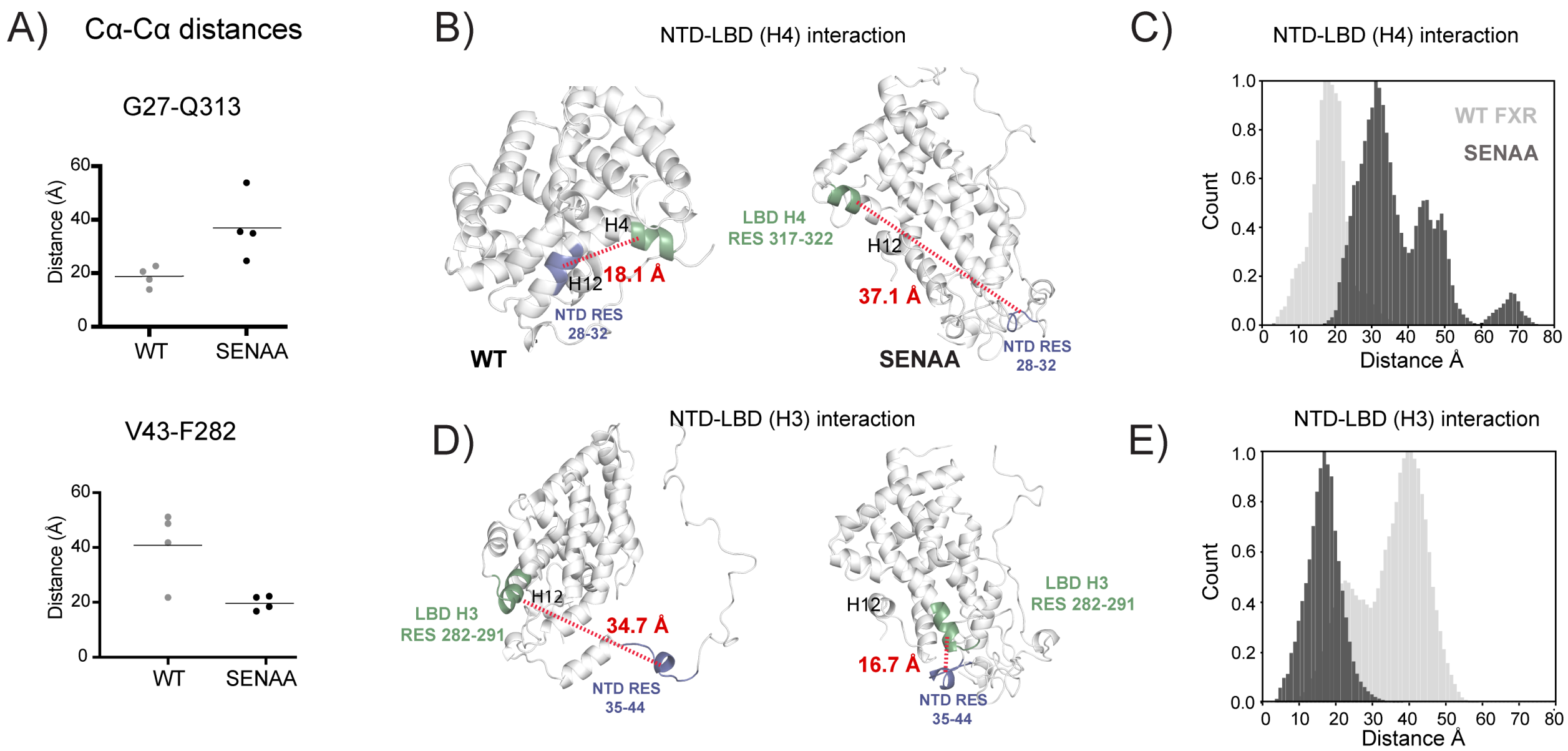

Figure S2. Effect of the SENAA mutation on NTD and LBD interactions. A, C-alpha distance between residue pairs G27-Q313 and V43-F282, illustrating a decrease and an increase in inter-residue distance upon SENAA mutation. B, NTD-LBD (H4) interaction, which weakens in the SENAA mutant. C, Histogram summarizing the NTD-LBD(H4) C-alpha distances, which are weakened in the SENAA mutant. D, NTD-LBD (H3) interaction, which is enhanced in the SENAA mutant. E, Histogram summarizing the NTD-LBD(H3) C-alpha distances, which are enhanced in the SENAA mutant.
